# Phyllosphere *Bacillus subtilis* Strain L-1 Enhances Resistance of Mango Leaves to *Colletotrichum* Infection

**DOI:** 10.3390/microorganisms14040906

**Published:** 2026-04-17

**Authors:** Yingfeng Niu, Xiaoping Zhu, Changli Mao, Xiaoran Qian, Ni Liu, Junmin Ai, Chenwanli Li, Jin Liu, Shuxing Liu

**Affiliations:** Molecular Biology Research Center, Yunnan Institute of Tropical Crops, Jinghong 666100, Xishuangbanna, China; niuyingfeng@163.com (Y.N.); zhu_xiaoping123@163.com (X.Z.); maokangwan@163.com (C.M.); mosaicclaw@163.com (X.Q.); liuni3710@163.com (N.L.); 18301612559@163.com (J.A.); 18800203519@163.com (C.L.)

**Keywords:** beneficial and pathogenic microbes, mango anthracnose, phyllosphere microbiota, *Bacillus*

## Abstract

Mango (*Mangifera indica* L.), a major tropical fruit crop, suffers severe anthracnose damage caused by *Colletotrichum* spp., and traditional chemical control has environmental and food safety risks, with plant-microbe interaction-based biological control as a sustainable alternative. However, the regulatory role of phyllosphere microbiota in the tripartite interactions among mango, beneficial microbes and *Colletotrichum* remains unclear. This study explored phyllosphere microbiota’s function in mango resistance to *Colletotrichum* and clarified the biocontrol mechanism of key beneficial isolates. We found *Colletotrichum* infection significantly reshaped mango leaf endophytic and epiphytic microbial communities, enriching *Burkholderia*, *Acinetobacter*, *Bacillus* and other dominant genera. We isolated a *B. subtilis* strain L-1 from the epiphytic microbiota that was 18-fold enriched in *Colletotrichum*-infected mango leaves. This strain exhibited potent antagonistic activity against *Colletotrichum siamense* with a relative inhibition rate of 82.10%, and delivered 79.77% biocontrol efficacy on mango leaves via two synergistic pathways: inhibiting pathogen spore germination and penetration by producing antimicrobial secreted metabolites and volatile organic compounds, and enhancing host disease resistance. Our findings advance the understanding of plant-phyllosphere microbiota-pathogen tripartite interactions and provide elite microbial resources for sustainable anthracnose management.

## 1. Introduction

Globally, mango ranks as the fifth most widely cultivated fruit by planting area, with China being the world’s second-largest mango-producing region. The mango industry plays a pivotal role in China’s agricultural economy [1]. In 2021, China’s mango cultivation area exceeded 374,600 hectares, with an annual output of 3.958 million tons, corresponding to a total output value of 21.14 billion yuan (RMB) [2]. However, mango diseases pose a severe threat to the industry, causing substantial economic losses. In China, at least 88 distinct mango diseases have been documented, among which anthracnose is the most devastating and prevalent [3]. Pathogens of anthracnose, primarily *Colletotrichum* spp., infect young mango leaves, shoots, inflorescences, and fruits, leading to leaf withering, abscission, and postharvest fruit rot, significantly hampering plant growth and fruit quality [4]. Specifically, *Colletotrichum* infection during the growing season can result in yield losses exceeding 10%, while postharvest infections during storage typically cause losses of 30–50%, and even up to 100% in severe cases [5].

Mango anthracnose is caused by fungi of the genus *Colletotrichum*, with *C. siamense*, *C. fructicola*, and *C. asianum* identified as the dominant pathogenic species in China [6]. Currently, chemical fungicides remain the primary control measure for mango anthracnose. Nevertheless, the long-term and excessive application of these fungicides has led to severe environmental pollution, the evolution of pathogen resistance, and ecological imbalances [7]. In contrast, biological control agents (BCAs) have gained considerable attention as promising alternatives to chemical fungicides due to their environmental friendliness, safety for non-target organisms, and low risk of inducing pathogen resistance [8,9].

The phyllosphere microbiota acts as a key biological barrier protecting host plants against pathogen invasion, and the structural and functional dynamics of this microbial community directly modulate plant health [10]. Accumulating evidence indicates that plants can regulate the restructuring of their phyllosphere microbiota, thereby enriching microbial taxa with antagonistic functions when challenged by pathogens [11]. For example, in the annual herbaceous vegetable cucumber, powdery mildew infection significantly reshapes the phyllosphere microbial community structure and drives a shift toward a disease-resistant microbiota [12].

Despite these findings, relevant investigations on pathogen-induced phyllosphere microbiome recruitment remain highly limited, with studies only conducted in the annual herbaceous plants. Perennial ligneous plants have coevolved in interaction with specific fungal and bacterial communities that differ significantly from those of annual plants, and their specificities in shaping functional microbial communities depend on high heterozygosis, physiological and molecular status associated with seasonality and aging processes, as well as their long-lived above-ground architectures [13]. Furthermore, perennial plants can live for decades or even centuries, and their continuous host-microbiome interactions may lead to gradual enrichment of specific microbial taxa that harbor functional traits associated with long-term disease suppression and systemic immunity-a dimension of microbial recruitment that is inherently absent in annuals [14]. Thus, while annual herbaceous plants provide valuable baseline insights, their findings cannot be directly extrapolated to perennial fruit trees without systematic validation.

To date, however, systematic investigations on the dynamic changes, functional mechanisms, and biocontrol potential of phyllosphere microbiota in perennial tropical fruit trees such as mango during pathogen infection are still insufficient, leaving a critical research gap in plant-microbiome-pathogen interactions. To address this knowledge gap, the present study investigated the structural discrepancies between endophytic and epiphytic microbiotas in healthy and *Colletotrichum*-infected mango leaves through the integration of functional microbial strain isolation and mechanistic analyses. The objectives of this study were to clarify the pivotal role of the phyllosphere microbiota in mango disease resistance; and lay a theoretical basis for the development of green control technologies based on the precise modulation of microbial communities.

## 2. Materials and Methods

### 2.1. Experimental Materials

‘Jinhuang’ mango leaves were used as experimental materials. Samples were collected from the Yunnan Innovation Base for Mango Germplasm Resources, Ministry of Agriculture and Rural Affairs, located in Jinghong City, Xishuangbanna Prefecture, Yunnan Province, China (22°1′12″ N, 100°47′15″ E). Fungal pathogens employed in this study, including *C. siamense*, *C. fructicola*, *Fusarium incarnatum*, and *Fusarium proliferatum*, were laboratory-preserved strains originally isolated from diseased mango leaves. Their ITS sequences have been deposited in GenBank under the following accession numbers: *C. siamense* PX248694, *C. fructicola* PZ247591, *F. incarnatum* PZ247592, and *F. proliferatum* PZ247593.

### 2.2. Culture Medium

Potato Dextrose Agar (PDA, BD Difco, Sparks, MD, USA) medium was prepared by dissolving 39 g/L PDA powder and 5 g/L agar in deionized water. Luria–Bertani (LB) broth was formulated with 10 g/L tryptone, 5 g/L yeast extract, and 10 g/L NaCl dissolved in deionized water, and the pH was adjusted to 7.4. The PDA-LA medium was prepared by adding appropriate amounts of PDA powder and agar to LB broth. All media were sterilized by autoclaving at 121 °C for 15 min prior to use.

### 2.3. Reagents and Instruments

All analytical-grade chemical reagents were purchased from Sinopharm Chemical Reagent Co., Ltd. (Shanghai, China). The 2× Phanta Max Master Mix was obtained from Vazyme Biotech Co., Ltd. (Nanjing, China). Primer synthesis and Sanger sequencing services were provided by Sangon Biotech (Shanghai) Co., Ltd. (Shanghai, China).

Key instruments included: autoclave (Systec GmbH, Linden, Germany); constant temperature incubator (Shanghai Yuejin Medical Instrument Co., Ltd., Shanghai, China); spectrophotometer (Shanghai Spectrum Instruments Co., Ltd., Shanghai, China); automated tissue lyser (QIAGEN GmbH, Hilden, Germany); and fluorescence microscope (LEICA Microsystems, Wetzlar, Germany).

### 2.4. Mango Phyllosphere Microbiome Analysis

Phyllosphere microbiota sample preparation was performed with minor modifications based on a previously reported method [15]. Briefly, phyllosphere microbiota samples were collected from light-green stage leaves of ‘Jinhuang’ mango plants, including both healthy plants and *Colletotrichum*-infected plants. Each experimental group included three independent biological replicates, with each replicate consisting of a minimum of 20 intact leaves. Rigorous measures were implemented to minimize cross-contamination between epiphytic and endophytic microbial fractions: fully independent, spatially separated workflows and dedicated disposable consumables were used for endophyte and epiphyte sample preparation throughout the entire process.

For endophytic microbiome analysis, leaves were surface-sterilized with 1% (*v*/*v*) ammonium hypochlorite solution for 1 min to thoroughly kill all epiphytic microorganisms attached to the leaf surface, followed by three consecutive 1 min rinses with sterile deionized water. This sequential washing step completely removed residual disinfectant, dead epiphytic microbial cells and extracellular DNA, ensuring no carryover contamination from the leaf surface into the endophytic fraction. The surface-sterilized leaves were then homogenized for endophytic DNA extraction.

For epiphytic microbiome analysis, only intact, undamaged leaves free of mechanical breakage were used to prevent tissue rupture and subsequent leakage of endophytic microorganisms, which would introduce false contamination into epiphytic samples. Separate batches of healthy and *Colletotrichum*-infected leaves were gently rinsed in pre-chilled sterile phosphate-buffered saline (PBS, pH 7.4) with constant shaking at 100 r/min for 1 h. After removing the leaves, the washing solution was centrifuged at 4500× *g* for 5 min at 4 °C, and the resulting microbial pellet was collected for epiphytic DNA extraction.

Genomic DNA was extracted from both endophytic and epiphytic samples. The V5-V7 and V3-V4 hypervariable regions of the 16S ribosomal RNA (rRNA) gene were amplified for endophytic and epiphytic samples, respectively. PCR amplicons were purified, recovered, and used for sequencing library construction. Paired-end sequencing was conducted on the Illumina MiSeq PE300 platform with a sequencing depth of 30,000 reads per sample. Raw sequence reads were subjected to quality filtering and adapter trimming, followed by Operational Taxonomic Unit (OTU) clustering, taxonomic annotation, alpha diversity analysis, genus-level composition analysis, beta diversity analysis, and differential abundance analysis.

### 2.5. Isolation and Identification of Dominant Phyllosphere Bacteria

Isolation and identification of dominant phyllosphere microorganisms were conducted with minor modifications according to the previously reported method [16]. *Colletotrichum*-infected ‘Jinhuang’ mango leaves at the light green stage were surface-sterilized and rinsed as described in Section 2.4, then cut into fine strips with sterile scissors. An appropriate volume of sterile distilled water was added to the leaf strips, and the mixture was homogenized using a tissue lyser. For epiphytic microbial isolation, separate diseased leaves were rinsed with sterile PBS to collect the epiphytic fraction.

Spore suspension of *C. siamense* (2 × 10^6^ conidia/mL) was mixed with an equal volume of either the leaf homogenate or epiphytic microbial suspension. The mixtures were evenly spread onto PDA-LA plates, air-dried in a laminar flow hood, and incubated at 28 °C for 5 days. Bacterial colonies forming clear inhibition zones against *C. siamense* were aseptically picked and sub-cultured in fresh LB broth for mass propagation.

Secondary screening was performed via the plate confrontation assay [17]. Briefly, 5 μL of a spore suspension of *C. siamense* (1 × 10^7^ conidia/mL) was spotted onto the center of a fresh PDA-LA plate. Four equidistant points (2.5 cm from the center) were inoculated with 5 μL of each candidate bacterial culture (OD_600_ = 0.8). Plates were air-dried and incubated at 28 °C for 5 days, after which the pathogen colony area was measured. The relative inhibition rate was calculated using the formula: Relative Inhibition Rate (%) = [(Sc − St)/Sc] × 100, where Sc represented pathogen colony area in control plates, and St represented pathogen colony area in test plates.

For bacterial identification, the 16S rRNA gene was amplified with primers 27F (5′-AGAGTTTGATCCTGGCTCAG-3′) and 1492R (5′-GGTTACCTTGTTACGACTT-3′), whereas the *rpoB* gene was amplified with primers rpoB-F (5′-AGGTCAACTAGTTCAGTATGGAC-3′) and rpoB-R (5′-AGAACCGTAACCGGCAACTT-3′) [18]. PCR amplicons were purified and subjected to Sanger sequencing. The resulting sequences were deposited into the GenBank database under accession numbers PX239505 (16S rRNA) and PX257355 (*rpoB*). Obtained sequences were blasted against the NCBI nucleotide database. Phylogenetic trees were constructed using the Neighbor-Joining (NJ) method in MEGA 11 software with 1000 bootstrap replicates to assess branch reliability.

### 2.6. Evaluation of Biocontrol Efficacy on Mango Leaves

Evaluation of biocontrol efficacy was carried out with minor modifications based on the protocol described previously [19]. Newly expanded leaves from mango shoots were collected, surface-sterilized with 1% (*v*/*v*) NaClO solution, and rinsed three times with sterile distilled water. Inoculation sites were created by puncturing the leaf surface with a 12-needle bundle. Each site was inoculated with 5 μL of one of three treatments, namely the negative control (LB broth), the positive control (a mixture of spore suspension of *C. siamense* at 1 × 10^7^ conidia/mL and LB broth), and the test group (a mixture of spore suspension of *C. siamense* at 1 × 10^7^ conidia/mL and bacterial suspension with an OD_600_ value of 0.8). Lesion areas were measured at 7 days post-inoculation (dpi), and the relative inhibition rate was calculated using the formula described in Section 2.5 (Sc represented mean lesion area of positive control; St represented mean lesion area of test group). At least 10 leaves were used per treatment group, and the experiment was performed with three biological replicates.

### 2.7. Detection of Inhibitory Effects of Secreted Metabolites and Volatile Organic Compounds (VOCs) on C. siamense

The agar well diffusion assay [20] was used to evaluate the inhibitory activity of secreted metabolites from target bacterial strains. Bacterial cultures were incubated for 24 h at 28 °C, then centrifuged at 8000 r/min for 10 min. The resulting supernatant was filter-sterilized through a 0.22 μm sterile filter membrane to obtain cell-free fermentation filtrates (CFFs). For each assay, 100 μL of spore suspension of *C. siamense* (1 × 10^7^ conidia/mL) was evenly spread onto PDA plates, which were then aseptically air-dried in a laminar flow hood. A sterile borer (15 mm diameter) was used to punch a single well at the center of each inoculated plate, and 150 μL of sterile CFF was immediately added to the well. Plates were incubated upright in an incubator at 28 °C for 24 h, after which the formation of inhibition zones was observed. The experiment was performed in triplicate, with a minimum of three technical replicates for each biological replicate.

The dual-plate co-cultivation method [19] was adopted to assess VOC-mediated inhibitory effects on *C. siamense*. A 100 μL aliquot of bacterial suspension (OD_600_ = 0.8) was evenly spread onto LB agar plates, which were incubated at 30 °C for 24 h to form uniform bacterial lawns. Separately, 5 μL of spore suspension of *C. siamense* (1 × 10^7^ conidia/mL) was spotted at the center of a PDA plate and aseptically air-dried. After drying, the PDA plate was paired face-to-face with a bacterial lawn plate, and the edge of the paired plates was hermetically sealed with Parafilm M to prevent VOC leakage. The assembled plates (LB plate at the bottom, PDA plate on top) were incubated at 28 °C for 5 days. Following incubation, the colony area of *C. siamense* was measured using ImageJ software (Version 1.8), and the relative inhibition rate was calculated as described before. The experiment was repeated three times, with at least three technical replicates per repetition.

### 2.8. Detection of the Effects of Dominant Phyllosphere Microorganisms and Their Secreted Metabolites or VOCs on the Spore Germination Process of C. siamense

Assays were performed with slight modifications based on previously reported protocols [21,22,23], using onion epidermal peels as a model system to simulate plant epidermal barriers. Onion epidermal peels were used as a standardized model system because they consist of a single layer of transparent, non-chlorophyllous cells that enable direct high-resolution visualization of fungal early infection processes, which cannot be clearly observed in thick, chlorophyll-rich mango leaves. To evaluate the effects of bacterial cells and secreted metabolites on *C. siamense* spore germination, onion epidermal peels (2 cm × 2 cm) were aseptically placed flat onto 1.5% water agar (WA) medium, and each peel was treated with 100 μL of one of three treatments, namely spore suspension of *C. siamense* (1 × 10^7^ conidia/mL, control), a mixture of *C. siamense*. spore suspension (1 × 10^7^ conidia/mL) and bacterial suspension (OD_600_ = 0.8), and a mixture of spore suspension of *C. siamense* (1 × 10^7^ conidia/mL) and CFFs. Plates were incubated at 28 °C for 7 h, and after incubation, spores on each epidermal peel were observed under a microscope; the germination rate was calculated by counting at least 300 spores per biological replicate, with germination defined as the emergence of a germ tube longer than half the width of the conidium. The experiment was conducted in triplicate, with at least three technical replicates for each biological replicate.

To evaluate the impact of bacterial VOCs on spore germination, 100 μL of spore suspension of *C. siamense* (1 × 10^7^ conidia/mL) was uniformly applied onto onion epidermal peels (2 cm × 2 cm) placed on 1.5% WA plates, followed by aseptic air-drying in a laminar flow hood. The WA plate was then paired face-to-face with either a sterile LB agar plate (negative control) or an LB agar plate with uniform bacterial lawns (test group) using the dual-plate co-cultivation method, and the paired plates were hermetically sealed with Parafilm M to prevent VOC leakage before being incubated at 28 °C for 7 h. Spore germination rate was determined via microscopic observation and counting as described before. Three independent experimental replicates were performed, and no fewer than three technical replicates were set in each replicate.

### 2.9. Detection of the Effects of Dominant Phyllosphere Microorganisms and Their Secreted Metabolites or VOCs on the Epidermal Penetration Process of C. siamense

Assays were conducted with minor modifications as described previously [24]. In order to evaluate the effects of bacterial cells and secreted metabolites on *C. siamense* epidermal penetration process, three 1 cm × 1 cm onion epidermal peel segments were aseptically placed flat on a single PDA plate, with each segment assigned to one of the three treatments listed in Section 2.8 (2 μL per segment). The PDA plate was incubated upright at 28 °C for 30 h to allow potential epidermal penetration by *C. siamense* spores, and after incubation, the epidermal peel segments were carefully removed with sterile forceps; the PDA plate was further incubated at 28 °C for an additional 36 h to promote the growth of *C. siamense* hyphae that had penetrated the epidermis. The larger colonies indicated more efficient epidermal penetration by the pathogen, whereas smaller colonies reflected suppressed penetration capacity. The experiment was performed three independent times, with at least five technical replicates in each independent experiment.

To assess the impact of bacterial VOCs on the epidermal penetration of *C. siamense*, two individual 35 mm-diameter PDA plates were each loaded with a 1 cm × 1 cm segment of onion epidermis; each segment was inoculated with 2 μL of spore suspension of *C. siamense* (1 × 10^7^ conidia/mL) and subsequently subjected to aseptic air-drying in a laminar flow hood. Each PDA plate was then paired face-to-face with either a sterile LA plate (negative control) or a bacterial lawn plate (test group) using the dual-plate co-culture method, and sealed tightly with Parafilm M. The assembled plates (LA plate at the bottom, PDA plate on top) were incubated at 28 °C for 30 h. After incubation, the epidermal peel segments and top LA plates were removed, and the PDA plates were further incubated at 28 °C for 36 h. Three independent experimental repetitions were conducted, each with at least five technical replicates.

### 2.10. Data Analysis

All data were processed in Microsoft Excel, and statistical analysis to determine significant differences between groups was conducted using GraphPad Prism 8. Data normality and homogeneity of variance were tested by the Shapiro–Wilk test and Levene’s test respectively before parametric analysis; Student’s *t*-test was used for pairwise comparisons of means between two groups, and one-way ANOVA followed by Tukey’s multiple comparison test was applied for comparisons among three or more groups.

## 3. Results

### 3.1. Colletotrichum Infection Caused Significant Alterations in the Endophytic Microbiota of Mango Leaves

**Figure 1:**
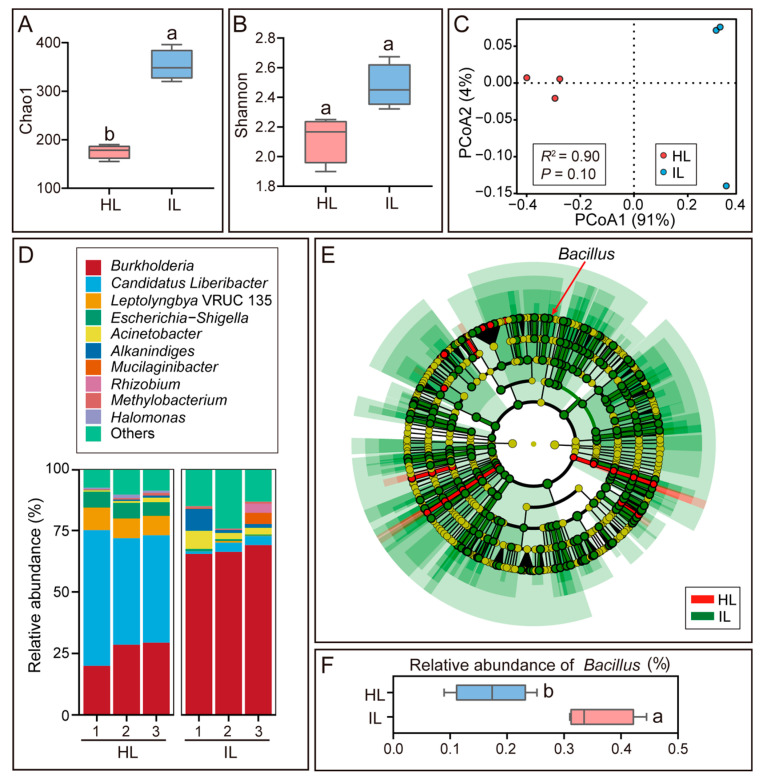
Analysis of the endophytic microbiota in healthy mango leaves (HL) and *Colletotrichum*-infected mango leaves (IL). (**A**) Chao1 richness index and (**B**) Shannon diversity index of the endophytic microbiota in HL and IL. (**C**) Principal Coordinates Analysis (PCoA) of the endophytic microbiota based on Bray–Curtis distances, showing differences between HL and IL. (**D**) Relative abundance-based taxonomic composition of the endophytic microbiota at the genus level in HL and IL. (**E**) Linear Discriminant Analysis Effect Size (LEfSe) cladogram of the endophytic microbiota. Concentric circles represent taxonomic levels from phylum (center) to genus (periphery). Red nodes: taxa significantly enriched in HL; green nodes: taxa significantly enriched in IL; yellow nodes: non-significant taxa. (**F**) Relative abundance of *Bacillus* in the endophytic microbiota between HL and IL. Data are presented as mean ± SEM. Different lowercase letters above the bars indicate statistically significant differences (*p* < 0.05, Student’s *t*-test).

To investigate the role of the phyllosphere microbiota in mango’s defense against *Colletotrichum* infection, the microbiomes of healthy mango leaves and those infected with anthracnose were analyzed. The Chao1 richness index of the endophytic microbiome was significantly higher in *Colletotrichum*-infected leaves than in healthy counterparts (*p* < 0.05), whereas no significant difference in the Shannon diversity index was observed between the two groups (Figure 1A,B). Principal Coordinates Analysis (PCoA) based on Bray–Curtis distances revealed substantial differences in the microbial composition of endophytic microbiomes between *Colletotrichum*-infected and healthy leaves (R^2^ = 0.90), with PCoA1 and PCoA2 accounting for 91% and 4% of the intergroup variance, respectively (Figure 1C).

A total of 26 phyla, 42 classes, 99 orders, 158 families, and 311 genera of bacteria were identified in the endophytic microbiota of both healthy and *Colletotrichum*-infected leaves. *Candidatus Liberibacter* was the most abundant bacterial genus in the endophytic microbiome of healthy leaves (47.41%), while its relative abundance decreased 17-fold to 2.85% in *Colletotrichum*-infected leaves. In contrast, *Burkholderia* became the dominant genus in infected leaves, with a relative abundance of 66.91% (Figure 1D, Table S1). Linear Discriminant Analysis Effect Size (LEfSe) analysis indicated that 267 microbial taxa, including the genera *Burkholderia*, *Pseudomonas*, and *Bacillus*, were significantly enriched in the endophytic microbiota of *Colletotrichum*-infected leaves (Figure 1E, Table S2).

### 3.2. Colletotrichum Infection Caused Significant Alterations in the Phyllosphere Epiphytic Microbiota of Mango Leaves

**Figure 2:**
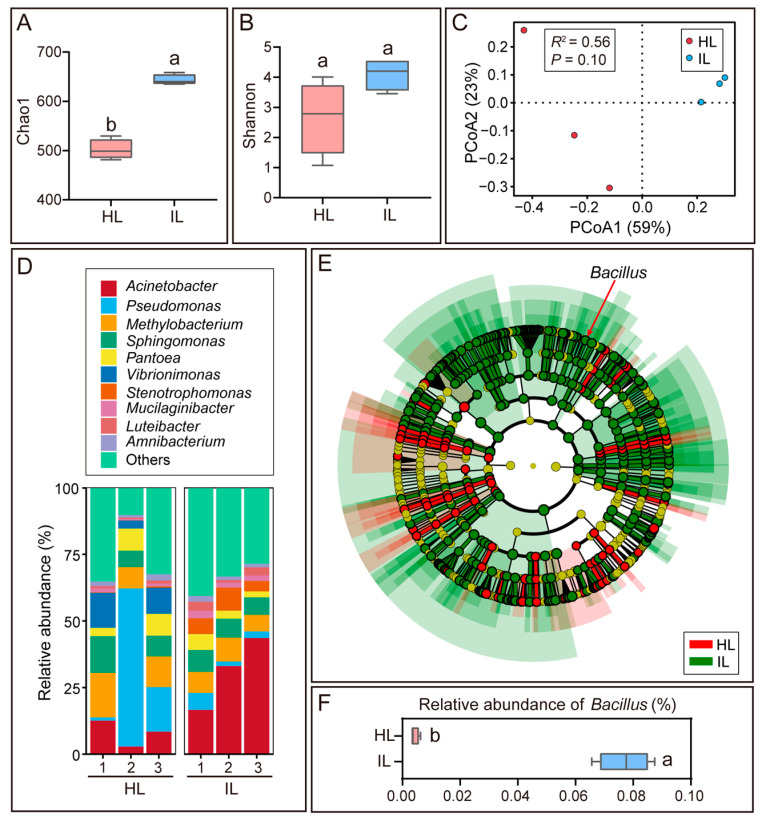
Analysis of the epiphytic microbiota in healthy mango leaves (HL) and *Colletotrichum*-infected mango leaves (IL). (**A**) Chao1 richness index and (**B**) Shannon diversity index of the epiphytic microbiota between HL and IL. (**C**) Principal Coordinates Analysis (PCoA) ordination of the epiphytic microbiota based on Bray–Curtis distances, showing differences between HL and IL. (**D**) Relative abundance-based taxonomic composition of the epiphytic microbiota at the genus level in HL and IL. (**E**) Linear Discriminant Analysis Effect Size (LEfSe) cladogram of the epiphytic microbiota. (**F**) Relative abundance of *Bacillus* in the epiphytic microbiota between HL and IL. Data are presented as mean ± SEM. Different lowercase letters above the bars denote statistically significant differences (*p* < 0.05, Student’s *t*-test).

In addition to the endophytic microbiota, the epiphytic microbiota, which constitutes the first line of defense against pathogen invasion, also exhibited marked changes in response to *Colletotrichum* infection. Consistent with the changes in the endophytic microbiota, the Chao1 richness index of the epiphytic microbiome was significantly higher in *Colletotrichum*-infected mango leaves than in healthy counterparts (*p* < 0.05), while no significant difference in the Shannon diversity index was detected between the two groups (Figure 2A,B). PCoA analysis based on Bray–Curtis distances revealed distinct differences in the microbial composition of epiphytic microbiomes between diseased and healthy leaves (R^2^ = 0.56), with PCoA1 and PCoA2 accounting for 59% and 23% of the intergroup variance, respectively (Figure 2C).

A total of 21 phyla, 46 classes, 101 orders, 162 families, and 337 genera of bacteria were identified in the epiphytic microbiota of both healthy and diseased leaves. *Pseudomonas* was the most abundant bacterial genus in the epiphytic microbiota of healthy leaves (25.74%), but its relative abundance declined to 3.51% in diseased leaves. In contrast, *Acinetobacter* became the most abundant genus in diseased leaves (33.10%), exhibiting a 4-fold increase in relative abundance compared to healthy leaves (Figure 2D, Table S3). LEfSe analysis showed that 381 microbial taxa, including *Acinetobacter*, *Stenotrophomonas*, and *Bacillus*, were significantly enriched in the epiphytic microbiome of diseased leaves (Figure 2E, Table S4).

### 3.3. Phyllospheric Bacillus Enhances Mango Leaf Resistance to Anthracnose

**Figure 3:**
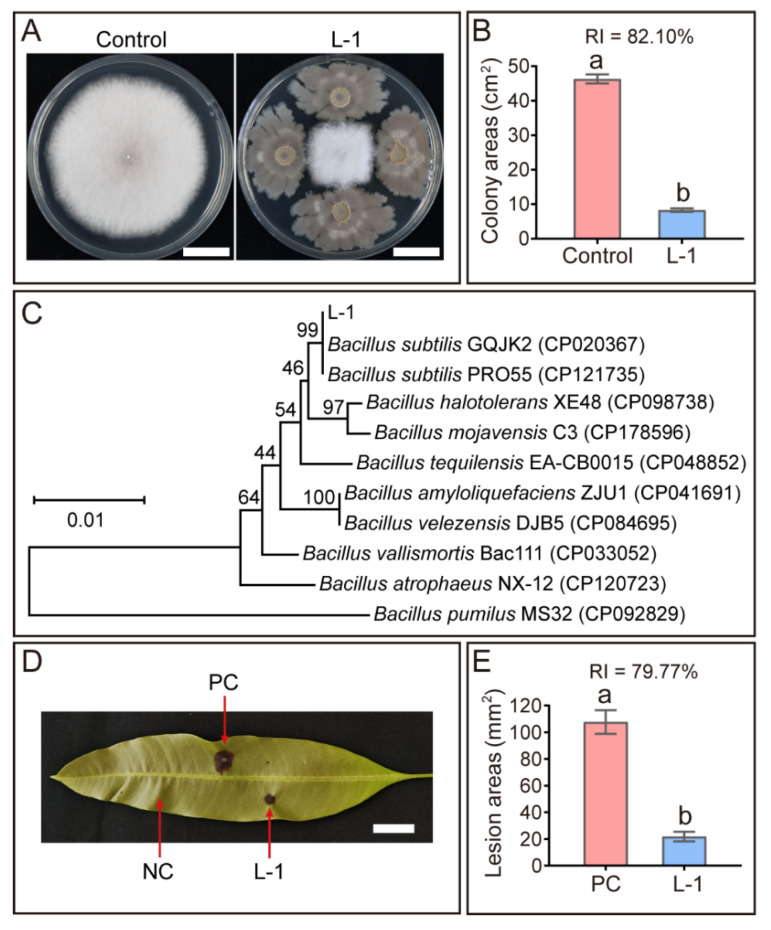
*Bacillus subtilis* strain L-1 enhances mango leaf resistance to anthracnose. (**A**) Colonies and (**B**) corresponding colony areas of *Colletotrichum siamense* co-cultured with or without strain L-1 on PDA-LA medium for 5 days. Scale bar = 2 cm. (RI) Relative inhibition rate. (**C**) Phylogenetic tree constructed based on concatenated sequences of the 16S rRNA and *rpoB* genes (Bootstrap = 1000 replicates). (**D**) Photographs and (**E**) lesion areas of mango leaves subjected to different inoculation treatments at 7 days post-inoculation. PC: positive control (a mixture of spore suspension of *C. siamense* and LB broth); NC: negative control (LB broth); L-1: a mixture of spore suspension of *C. siamense* and L-1 bacterial suspension. Scale bar = 2 cm. Data are presented as mean ± SEM. Different lowercase letters above the bars denote statistically significant differences (*p* < 0.05, Student’s *t*-test).

To identify dominant phyllospheric microbes involved in mango leaf defense against *Colletotrichum* infection, antagonistic bacteria exhibiting inhibitory activity against *C. siamense* were isolated from both endophytic and epiphytic microbiomes of *Colletotrichum*-infecte*d* mango leaves. A total of 248 bacterial strains were isolated, among the isolates, a bacterial strain designated L-1, isolated from the epiphytic microbiota of diseased leaves, exhibited strongest inhibitory activity against *C. siamense* growth, with a relative inhibition rate of 82.10% (Figure 3A,B). Accordingly, this strain was selected for further in-depth investigation. Combined morphological and molecular characterization confirmed this strain as *B. subtilis* (Figure 3C).

Assessment of the biocontrol efficacy of *B. subtilis* strain L-1 against *C. siamense* on mango leaves showed that it significantly reduced the lesion area induced by pathogen infection (*p* < 0.05), achieving a relative inhibition rate of 79.77% (Figure 3D,E). These results demonstrate that *B. subtilis* strain L-1 enhances mango leaf resistance to anthracnose. Consistently, the relative abundance of *Bacillus* in the endophytic and epiphytic microbiota of *Colletotrichum*-infected leaves was 2-fold and 18-fold higher than that in healthy leaves, respectively (Figure 1F and Figure 2F).

### 3.4. B. subtilis Strain L-1 Inhibits the Colletotrichum Infection Process by Producing Secreted Inhibitory Metabolites

**Figure 4:**
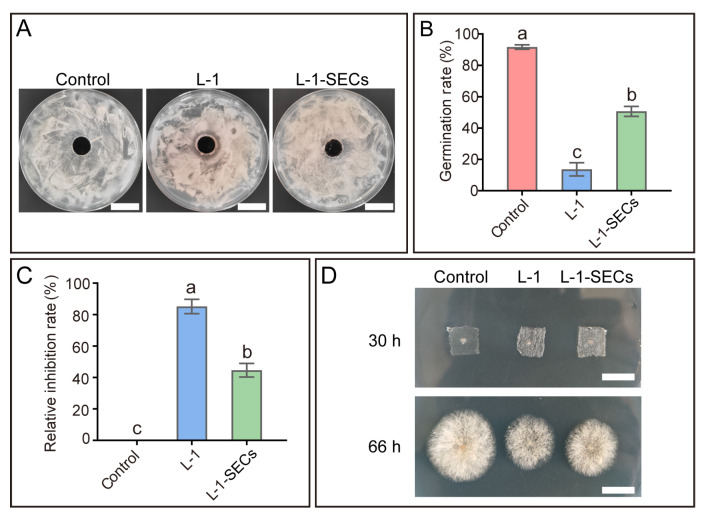
*Bacillus subtilis* strain L-1 inhibits the infection process of *Colletotrichum siamense* via the production of secreted antimicrobial compounds. (**A**) Growth inhibition of *C. siamense* following 24 h treatment with secreted antimicrobial compounds from strain L-1 (L-1-SECs). Scale bar = 2 cm. (**B**) Germination rate of *C. siamense* spores inoculated on onion epidermal peels at 7 h post-inoculation (hpi). (**C**) Relative inhibition rates of *B. subtilis* strain L-1 and its secreted antimicrobial compounds (L-1-SECs) against *C. siamense* spore germination. (**D**) Effects of *B. subtilis* strain L-1 and L-1-SECs on the epidermal penetration efficiency of *C. siamense*. Scale bar = 1 cm. Upper panel: *C. siamense* on onion epidermal peels at 30 hpi. Lower panel: Hyphal growth of *C. siamense* that penetrated the epidermis, following removal of the onion epidermal peels and an additional 36 h incubation. Data are presented as mean ± SEM. Different lowercase letters above the bars indicate statistically significant differences (*p* < 0.05, Tukey’s test in one-way ANOVA).

To further elucidate the antagonistic mechanism of *B. subtilis* strain L-1 against *C. siamense*, its inhibitory activity was evaluated using cell-free fermentation filtrates (CFFs). Notably, the CFFs of strain L-1 exhibited moderate inhibitory activity against *C. siamense* growth (Figure 4A), indicating that strain L-1 antagonizes *C. siamense* by secreting inhibitory metabolites. Spore germination is the initial step of *Colletotrichum* infection in mango leaves [5]. After 7 h of inoculation on onion epidermal peels, 91.67% of *C. siamense* spores germinated in the control group, compared to only 13.67% in the group co-inoculated with strain L-1; treatment with CFFs from strain L-1 reduced the spore germination rate to 50.67% (Figure 4B). The relative inhibition rates of strain L-1 and its CFFs against *C. siamense* spore germination were 85.13% and 44.59%, respectively (Figure 4C). Epidermal penetration is a prerequisite for the successful internal colonization of plants by *Colletotrichum* [5]. Treatment with either *B. subtilis* strain L-1 or its secreted metabolites significantly suppressed the epidermal penetration efficiency of *C. siamense* (Figure 4D), confirming their inhibitory effects on the infection process of *C. siamense*.

### 3.5. B. subtilis Strain L-1 Inhibits the Colletotrichum Infection Process by Producing Volatile Inhibitory Compounds

**Figure 5:**
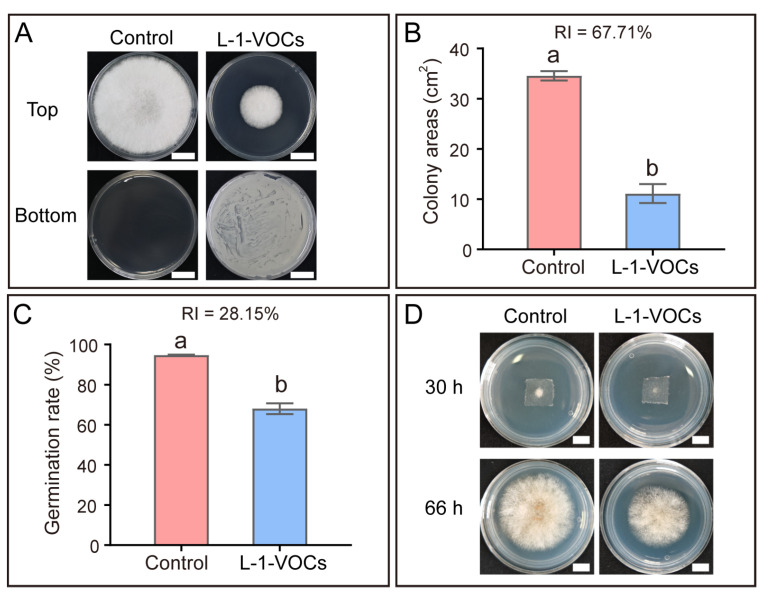
*Bacillus subtilis* strain L-1 inhibits the infection process of *Colletotrichum siamense* via the production of volatile antimicrobial compounds. (**A**) Colony morphology and (**B**) corresponding colony areas of *C. siamense* following 5 days of confrontation culture with strain L-1. The upper part of the photograph shows *C. siamense* at the top, while the lower part displays either the blank control or the strain L-1 at the bottom. Scale bar = 2 cm. L-1-VOCs: volatile organic compounds from strain L-1. (**C**) Spore germination rate of *C. siamense* at 7 h post-treatment with L-1-VOCs. (**D**) Effect of L-1-VOCs on the epidermal penetration efficiency of *C. siamense*. Scale bar = 5 mm. Data are presented as mean ± SEM. Different lowercase letters above the bars indicate statistically significant differences (*p* < 0.05, Student’s *t*-test).

In addition to secreted metabolites, VOCs represent another important class of antimicrobial substances produced by *Bacillus* spp. [19]. VOCs produced by *B. subtilis* strain L-1 exhibited significant inhibitory activity against the mycelial growth of *C. siamense*. Specifically, treatment with L-1 VOCs significantly reduced the colony area of *C. siamense* (*p* < 0.05), with a relative inhibition rate of 67.71% (Figure 5A,B). Furthermore, the VOCs of strain L-1 suppressed the spore germination of *C. siamense*. At 7 h post-inoculation, the spore germination rate of *C. siamense* reached 94.67% in the control group, whereas treatment with L-1 VOCs decreased the germination rate to 68.00%, corresponding to a relative inhibition rate of 28.15% (Figure 5C). Moreover, exposure to L-1 VOCs significantly suppressed the epidermal penetration efficiency of *C. siamense* (Figure 5D).

### 3.6. B. subtilis Strain L-1 Exhibits Broad-Spectrum Antagonism Against Mango Foliar Pathogenic Fungi

**Figure 6:**
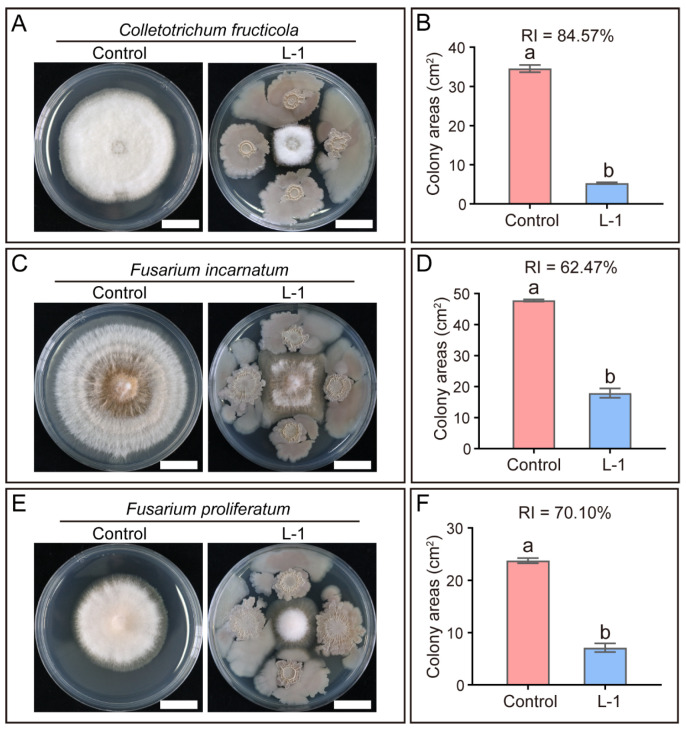
*Bacillus subtilis* strain L-1 exhibits broad-spectrum antifungal activity against mango foliar fungal pathogens. (**A**) Colony morphology and (**B**) corresponding colony areas of *Colletotrichum fructicola* following 7 days of dual-culture with strain L-1 on PDA-LA medium. Scale bar = 2 cm. (**C**) Colony morphology and (**D**) corresponding colony areas of *Fusarium incarnatum* following 7 days of dual-culture with strain L-1 on PDA-LA medium. Scale bar = 2 cm. (**E**) Colony morphology and (**F**) corresponding colony areas of *Fusarium proliferatum* following 7 days of dual-culture with strain L-1 on PDA-LB medium. Scale bar = 2 cm. Data are presented as mean ± SEM. Different lowercase letters above the bars indicate statistically significant differences (*p* < 0.05, Student’s *t*-test).

Beyond its activity against *C. siamense*, a broad-spectrum antagonistic profile is a highly desirable trait for biocontrol agents intended for field application. In addition to its potent activity against *C. siamense*, mango leaves are vulnerable to infection by multiple foliar pathogenic fungi. In this study, *B. subtilis* strain L-1 exhibited strong antagonistic activity against three other common mango foliar pathogens, namely *C. fructicola*, *F. incarnatum*, and *F. proliferatum*. The relative inhibition rates of strain L-1 against these pathogens were 84.57%, 62.47%, and 70.10%, respectively (Figure 6). These results confirm that *B. subtilis* strain L-1 possesses broad-spectrum antagonistic activity against mango foliar pathogenic fungi.

## 4. Discussion

The tripartite interactions among plants and their associated beneficial and pathogenic microbes have attracted increasing attention in recent years [25,26,27]. This study systematically elucidates the synergistic response mechanisms of the phyllosphere endophytic and epiphytic microbiomes in mango during anthracnose development. Notably, the relative abundance of *Bacillus* was significantly increased in both the endophytic and epiphytic microbiomes of *Colletotrichum*-infected leaves (Figure 1F and Figure 2F), indicating that mango plants enrich *Bacillus* to defend against *Colletotrichum* infection. This phenomenon may align with the plant “Cry for Help” theory, which posits that plants release specific metabolites (e.g., jasmonic acid and volatile organic compounds) upon pathogen attack to selectively recruit beneficial microorganisms [28]. For instance, after being infected by *Pseudomonas syringae*, *Arabidopsis thaliana* induces the secretion of L-malic acid in roots, which selectively signals and recruits the beneficial rhizobacterium *B. subtilis* in a dose-dependent manner [29]; rice plants recruit *Pseudomonas* spp. via the production of 4-hydroxycinnamic acid, thereby effectively suppressing bacterial blight outbreaks [30]. Alternatively, diseased plants with weakened defense systems may be more easily colonized by opportunistic and saprophytic microbes, leading to the enrichment of specific microbial taxa, and the increased microbial diversity detected in diseased mango trees also reflects the reduced resistance of infected leaves to microbial colonization. In this context, it is worth noting that screening biocontrol bacteria from healthy mango trees within a diseased orchard is a more optimized strategy for obtaining efficient antagonistic strains. Healthy plants that remain asymptomatic under disease pressure are more likely to harbor core beneficial microbial taxa that play a key role in host disease resistance, and strains isolated from these niches usually exhibit stronger adaptability to the host phyllosphere and more stable biocontrol efficacy. This perspective provides an important optimization direction for the high-throughput screening of anthracnose biocontrol agents in subsequent studies.

In addition to *Bacillus*, our microbiome analysis also revealed significant enrichment of *Burkholderia* and *Acinetobacter* in diseased leaves, which may also contribute to mango disease resistance. *Burkholderia* is a well-recognized plant growth-promoting rhizobacterium (PGPR) that colonizes both the rhizosphere and root interior. It enhances plant growth through nitrogen fixation, phosphate solubilization, and biosynthesis of auxins and siderophores, while also inhibiting the growth of diverse fungal pathogens via the production of antimicrobial substances [31,32,33]. *Acinetobacter* represents another important PGPR, which promotes plant growth by synthesizing auxins, siderophores, and gibberellins, as well as solubilizing phosphorus, potassium, and zinc. This genus also suppresses phytopathogenic fungi (e.g., *Fusarium graminearum*) through the secretion of antimicrobial compounds [34,35,36]. The substantial enrichment of these two genera in the phyllosphere of diseased mango leaves potentially aids host resistance to *Colletotrichum* infection either through direct pathogen inhibition or by promoting leaf growth. Nevertheless, this hypothesis requires validation through targeted experimental evidence.

When compared with previously reported biocontrol agents against mango anthracnose, strain L-1 exhibits comparable antagonistic efficacy (82.10%) and unique advantages as a native phyllosphere isolate. A range of biocontrol agents targeting *Colletotrichum* have been isolated from various environments, including *B. amyloliquefaciens* strain YM-11-C, *B. subtilis* strain N-16-2, *B. velezensis* strain RL-LL04 and L18-7, with relative inhibition rates against *Colletotrichum* spp. of 78.22%, 79.63%, 82.20%, and 83.00%, respectively [18,37,38]. As a native phyllosphere isolate from mango leaves, strain L-1 may have stronger adaptability and colonization ability on mango phyllosphere, which could be more conducive to field application and persistent disease control compared with exogenous biocontrol strains [39]. Recently, *B. subtilis* has also been used to control spinach leaf spot caused by *Alternaria alternata* [40], showing good biocontrol potential and supporting the application value of *Bacillus* species in the biological control of plant foliar diseases.

Regarding the mechanisms of action, our results demonstrate that strain L-1 exerts its biocontrol activity through dual pathways involving both secreted and volatile antimicrobial compounds. Extensive research has been conducted on the mechanisms of action of biocontrol agents against mango anthracnose. Some agents directly inhibit *Colletotrichum* via the production of antimicrobial substances: for example, *B. velezensis* RL-LL04 synthesizes volatile inhibitory compounds (e.g., benzaldehyde, 3-methylbutanoic acid, and phenol) to suppress pathogen growth [18], whereas *Streptomyces malaysiensis* HSL-9B secretes 12-methyltridecanoic acid as an antifungal agent [41]. Beyond direct antagonism, other biocontrol agents enhance mango resistance to anthracnose by activating systemic immunity or promoting plant growth. *B. amyloliquefaciens* GSBa-1 induces H_2_O_2_ accumulation and stimulates the synthesis of antifungal phenolic acids in mango fruit, thereby enhancing anthracnose resistance [42]; *Leclercia adecarboxylata* strain MHA-2-F1 promotes root development and plant growth by supplying nutrients (e.g., phosphorus, calcium, and iron) and producing auxins, which indirectly improves anthracnose resistance [43]. Although strain L-1 suppresses *C. siamense* through the combined action of secreted metabolites and VOCs, the identity of the key compounds mediating this antagonistic effect has not yet been elucidated and requires comprehensive investigation.

This study provides the first systematic characterization of phyllosphere bacterial community responses to *Colletotrichum* infection in mango, identifying *Bacillus* as a key disease-associated taxon and laying a foundation for microbiome-based biocontrol of mango anthracnose. Nevertheless, we have not yet conducted in vivo experiments to evaluate the effects of strain L-1 on plant growth promotion and disease resistance using mango seedlings. Furthermore, all experiments were conducted under controlled laboratory conditions, and the findings await further field validation across diverse mango cultivars, growth stages and geographical regions. In addition, the active antimicrobial metabolites produced by strain L-1 have not yet been identified and purified, and the taxonomic classification of strain L-1 in this study was only based on 16S rRNA and *rpoB* gene sequences, which has certain limitations for accurate species-level identification. Future studies will further validate strain L-1 and refine the biocontrol system for mango anthracnose. Greenhouse pot experiments with mango seedlings will first be performed to evaluate its plant growth-promoting and anthracnose suppression efficacy, followed by large-scale field validation across diverse cultivars, growth stages and geographical regions. Meanwhile, whole-genome sequencing of strain L-1 will be conducted in our follow-up work to complete the accurate taxonomic classification of the strain. Subsequent work will focus on isolating and characterizing its active antimicrobial metabolites, and elucidating the molecular mechanisms underlying its biocontrol and growth-promoting activities.

## 5. Conclusions

*Colletotrichum* infection enriches specific microorganisms, including *Burkholderia*, *Acinetobacter*, and *Bacillus*, within their endophytic and epiphytic microbiota in mango leaves. *B. subtilis* strain L-1, isolated from the leaf epiphytic microbiome, exhibited potent inhibitory efficacy against *C. siamense* on both artificial culture medium and mango leaves. Strain L-1 produced both secreted and volatile inhibitory compounds, thereby promoting mango leaves to resist pathogen infection by inhibiting *C. siamense* spore germination and epidermal penetration.

## Data Availability

The original data presented in the study are openly available in the GenBank database under the BioProject accession number PRJNA1434091 (corresponding Sequence Read Archive accessions: SRR37527990, SRR37527989, SRR37527978, SRR37527973, SRR37527972, SRR37527971, SRR37527970, SRR37527969, SRR37527968, SRR37527967, SRR37527988, SRR37527987, SRR37527986, SRR37527985, SRR37527984, SRR37527983, SRR37527982, SRR37527981, SRR37527980, SRR37527979, SRR37527977, SRR37527976, SRR37527975, SRR37527974). The 16S rRNA and rpoB gene sequences have also been deposited in GenBank with the respective accession numbers PX239505 and PX257355.

## Supplementary Materials

The following supporting information can be downloaded at: https://www.mdpi.com/article/10.3390/microorganisms14040906/s1, Table S1: Relative abundance of each genus in the endophytic microbiota of mango leaves; Table S2: Biomarkers in mango leaf endophytic microbiota identified by LEfSe analysis; Table S3: Relative abundance of each genus in the epiphytic microbiota of mango leaves; Table S4: Biomarkers in mango leaf epiphytic microbiota identified by LEfSe analysis.
